# EGCG inhibited bladder cancer T24 and 5637 cell proliferation and migration via PI3K/AKT pathway

**DOI:** 10.18632/oncotarget.24301

**Published:** 2018-01-16

**Authors:** Ke-Wang Luo, Wing-Yin Lung, Chun-Xie, Xin-Le Luo, Wei-Ren Huang

**Affiliations:** ^1^ Key Laboratory, People’s Hospital of Longhua, Shenzhen, China; ^2^ Key Laboratory of Medical Programming Technology, Shenzhen Second People’s Hospital, The First Affiliated Hospital of Shenzhen University, Shenzhen, China

**Keywords:** EGCG, bladder cancer, T24, 5637, PI3K/AKT

## Abstract

Epigallocatechin-3-gallate (EGCG), the bioactive polyphenol in green tea, has been demonstrated to have various biological activities. We previously found that EGCG inhibited SW780 tumor growth by down-regulation of NF-κB and MMP-9. This study demonstrated that EGCG inhibited bladder cancer T24 and 5637 cell proliferation and migration via PI3K/AKT pathway, without modulation of NF-κB. Our results showed that treatment of EGCG resulted in significant inhibition of cell proliferation by induction of apoptosis, without obvious toxicity to normal bladder SV-HUC-1 cells. EGCG also inhibited 5637 and T24 cell migration and invasion at 25–100 μM. Western blot confirmed that EGCG induced apoptosis in T24 and 5637cells by activation of caspases-3 and PARP. Besides, EGCG up-regulated PTEN and decreased the expression of phosphorylated PI3K, AKT in both T24 and 5637 cells. In addition, animal study demonstrated that EGCG (100 mg/kg, i.p. injected daily for 4 weeks) decreased the tumor weight in mice bearing T24 tumors by 51.2%, as compared with the untreated control. EGCG also decreased the expression of phosphorylated PI3K and AKT in tumor, indicating the important role of PI3K/AKT in EGCG inhibited tumor growth. When AKT was inhibited, EGCG showed no obvious effect in cell migration in T24 and 5637 cells. In conclusion, our study elucidated that EGCG was effective in inhibition of T24 and 5637 cell proliferation and migration, and presented evidence that EGCG inhibited cell proliferation and tumor growth by modulation of PI3K/AKT pathway.

## INTRODUCTION

Bladder cancer is one of the most common cancers among people, which ranks 9th of world cancer and caused 165,000 deaths in 2012 [1]. There are about 430 000 new cases of bladder cancer globally in 2012, and about 10,063 new cases in the UK in 2014 [2]. Globally, bladder cancer resulted in 170,000 deaths in 2010 [3]. Therefore, bladder cancer still takes a tremendous toll. Although many significant advances on the frontline bladder cancer research and chemotherapy have been developed, the efficacies of current therapies are limited by a range of adverse effects, toxicity and drug resistance. Therefore, novel therapeutic strategies and more effective agents for advanced disease are still urgently needed.

Epigallocatechin-3-gallate (EGCG), the most abundant and bioactive polyphenol in green tea, is demonstrated to have various biological activities, including cardiovascular protection, anti-obesity and anti-cancer effects [4]. Epidemiological studies have shown that tea polyphenol or EGCG intake was associated with increased weight loss due to diet-induced thermogenesis [5]. Recent data from clinical and research indicated that EGCG consumption may help reducing the risk of cardiovascular diseases and then lower the rate of heart diseases [6]. Besides, there are a number of literatures reported the anti-cancer effect of EGCG. It was demonstrated that treatment of EGCG resulted in significant inhibition of tumor growth, and significant reduction of growth factor of EGFR and IGF in serum in prostate and breast cancers [7–8]. EGCG was effective in lowering the risk of several cancer types, including stomach, prostate and lung cancers through inducing apoptosis, inhibition of metastasis and angiogenesis [9]. Recently, varieties of studies investigated the effects of EGCG in bladder cancer. *In vitro* studies demonstrated that EGCG inhibits cell proliferation and migration in bladder cancer T24, MBT-2, TCCSUP and SW780 cells [10–12]. Kemberling et al. elucidated that intravesical treatment of EGCG resulted in the lowering risk of AY-27 tumor by 64% in rats as compared with untreated control [13]. Clinical study showed that bladder cancer patients received tea polyphenol including EGCG resulted in a decreasing level of PCNA which is related to cell proliferation and metastasis [14]. In addition, our previous study elucidated that EGCG was effective in inhibition SW780 cell proliferation and migration, and EGCG inhibited SW780 tumor growth by down-regulation of NF-κB and MMP-9 [15]. Bladder cancer T24 and 5637 cells are in the advanced stage of human bladder cancer, with high capacity of proliferation and metastasis. This study aimed to investigate whether EGCG is effective in inhibition of T24 and 5637 cell proliferation and migration both *in vitro* and *in vivo*.

During the process of cancer propagation, PI3K/AKT pathway plays an important role. Over activation of the AKT pathway can promote cell proliferation and migration [16]. Recent studies demonstrated that over activation of PI3K/AKT signaling pathway could promote the occurrence and development of bladder cancer. It was demonstrated that about 94% advanced bladder cancer patients showed significantly decrease of tumor suppressor gene PTEN [17]; when the intracellular level of PTEN was improved by transfection of adenovirus vector, the bladder cancer cell growth was inhibited and induced apoptosis [18]. Oka et al. found that activation of AKT can prevent the apoptosis in bladder cancer T24 cells. However, when the inhibitor Wortmannin was added, the AKT expression was down-regulated and then inhibited bladder cancer cell proliferation [19].

The present study aimed to investigate the anti-cancer effect of tea polyphenol EGCG in bladder cancer T24 and 5637 cell lines both *in vitro* and *in vivo*. Also, the role of EGCG in different mechanisms of action would be discussed. Here, we assessed the apoptosis-induction, anti-migration and anti-invasion abilities of EGCG in T24 and 5637 cells *in vitro*, and then further evaluated the anti-tumor activities of EGCG in nude mice bearing T24 tumors. Besides, the involvement of PI3K and AKT were also evaluated both *in vitro* and *in vivo* after EGCG treatment.

## RESULTS

### EGCG inhibited bladder cancer T24 and 5637 cell proliferation

**Figure 1:**
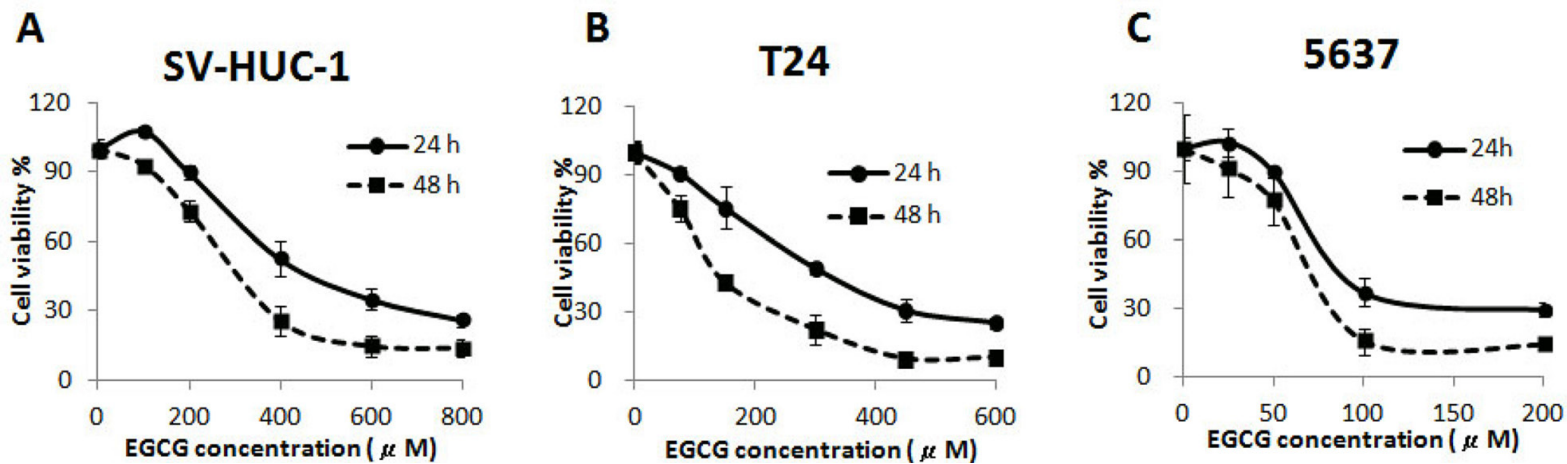
Cytotoxicity of EGCG on normal bladder epithelium SV-HUC-1 (**A**) and bladder cancer T24 (**B**) and 5637 (**C**) cells, after 24 and 48 h incubation. Data were expressed as mean ±SD.

Treatment with EGCG for 24 and 48 h resulted in inhibition of cell proliferation in a time- and dose-dependent manner bladder cancer T24 and 5637 cells. As shown in Figure 1, EGCG inhibited the growth of T24 and 5637 cells with an IC_50_ of 117.8, 69.5 μM at 48 h, respectively. Besides, the cytotoxicity of EGCG on normal human bladder epithelium SV-HUC-1 cells was also tested. The results showed that EGCG was much more sensitive in bladder cancer cells (T24, 5637) than in SV-HUC-1 cells. EGCG inhibited the growth of SV-HUC-1 cells with an IC_50_ of 317.2 μM at 48 h, which was much higher than that in T24 and 5637 cells (Figure 1C).

### EGCG induced apoptosis in T24 and 5637 cells

**Figure 2:**
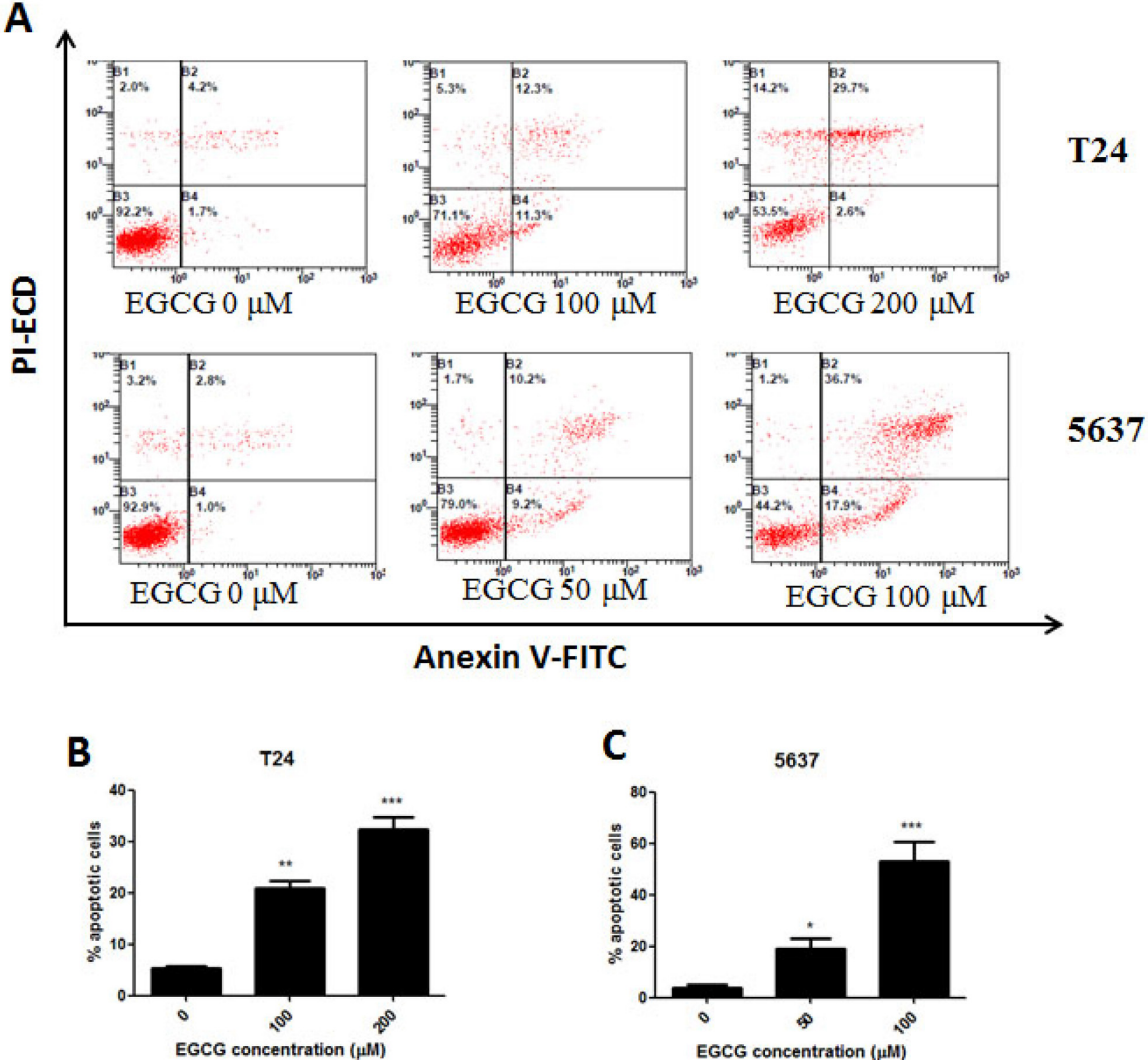
Induction of apoptosis on T24 and 5637 cells by EGCG (**A**) Flow cytometry images. (**B**–**C**) Quantitative analysis of the percentage of apoptotic cells of EGCG on T24 (B) and 5637 (C) cells after 24 h incubation .The percentage of total apoptotic cells was defined as the sum of early and late apoptotic cells. Data were presented as mean + SD (*n* = 3). ^*^p < 0.05, ^**^p < 0.01 and ^***^p < 0.001, as compared with untreated control.

Annexin-V FITC/PI staining was performed to determine whether EGCG induced apoptosis in bladder cancer cells. The results showed that when T24 and 5637 cells were incubated with increasing dose of EGCG, the rates of cell apoptosis were increased in a dose-dependent manner. The percentage of apoptotic cells upon treatment with 100 and 200μM of EGCG in T24 cells were found to be 21.8% and 32.5% after 24 h incubation (Figure 2). Treatment with 50 and 100μM of EGCG in 5637 cells caused 19.8% and 54.2% apoptotic cells (Figure 2A, 2C).

### EGCG inhibited cell migration and invasion in T24 and 5637 cells

**Figure 3:**
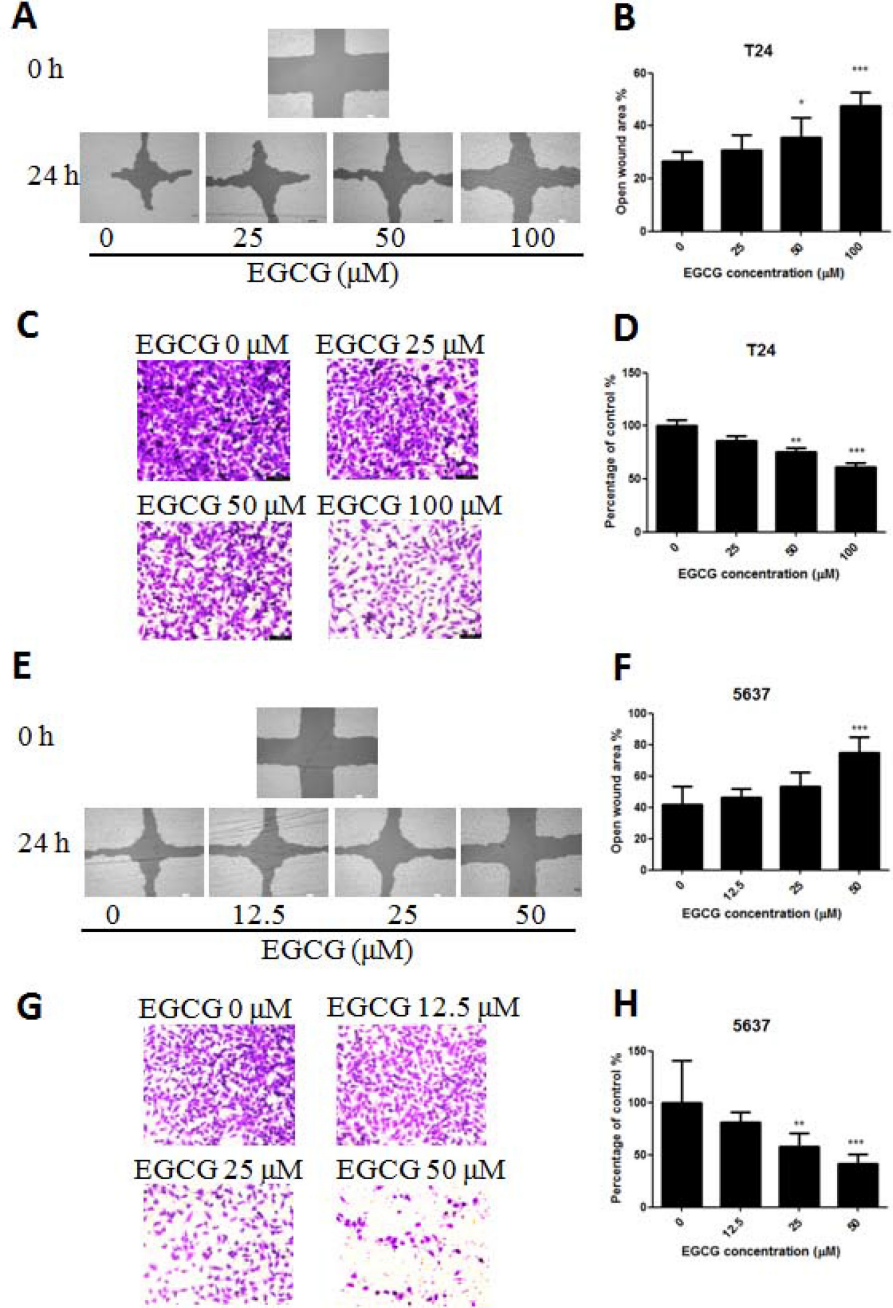
Effect of EGCG on T24 and 5637 cell migration and invasion activities (**A**, **E**) Representative images of the wounded cell monolayers of T24 (A) and 5637 (E) cells. (**B**, **F**) Quantitative analysis of the anti-migration activity of EGCG on T24 (B) and 5637 (F) cells after 24 h incubation. Data were expressed as the percentage of open wound area from baseline cultures without treatment. (**C**, **G**) Representative images of the stained T24 (C) and 5637 (G) cells. (**D**, **H**) Quantitative analysis of the anti-invasion activity of EGCG on T24 (D) and 5637 (H) cells. Data were presented as mean + SD (*n* = 3). ^*^p < 0.05, ^**^p < 0.01 and ^***^p < 0.001, as compared with untreated control.

To determine the efficacy of EGCG against bladder cancer cell metastasis *in vitro*, the scratch wound and transwell migration assays were introduced. As shown in Figure 3A, EGCG significantly inhibited T24 cell migration from 25 μM after 24 h incubation, and the inhibition was enlarged when the concentration increased (Figure 3B). Besides, the result from transwell migration assay was in line with the data from scratch assay. In Figure 3C, EGCG inhibited T24 cell invasion efficiently with the increase of EGCG concentration. In the presence of 50 and 100 μM, EGCG inhibited cell invasion of SW780 cells significantly by 25.2% and 37.7%, respectively (Figure 3D). Similar results were also found in 5637 cells, EGCG inhibited 5637 cell migration and invasion in a dose dependent manner, and significant differences were shown between EGCG (50 μM) and control (Figure 3E–3F).

### EGCG regulated the protein expressions

**Figure 4:**
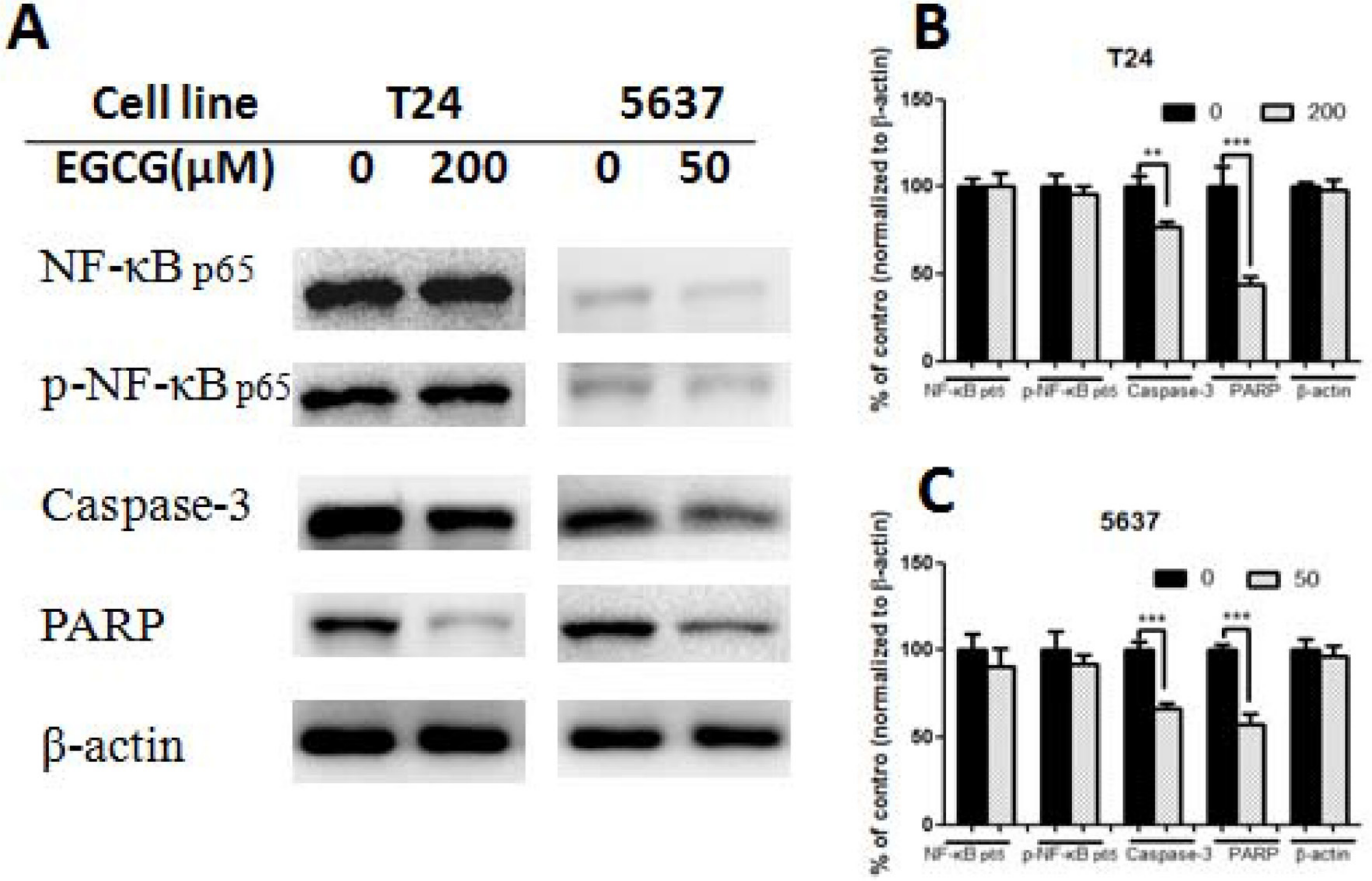
Effect of EGCG on protein expression (**A**) Representative images of western blot of proteins after treated with EGCG in T24 and 5637 cells. (**B**–**C**) Statistical analysis of NF-κB p65, phosphorylated NF-κB p65, caspase-3, PARP and β-actin protein expressions in T24 (B) and 5637 (C) cells after EGCG treatment. Data were showed as mean + SD (*n* = 3). ^**^p < 0.01 and ^***^p <0.001, as compared with untreated control.

**Figure 5:**
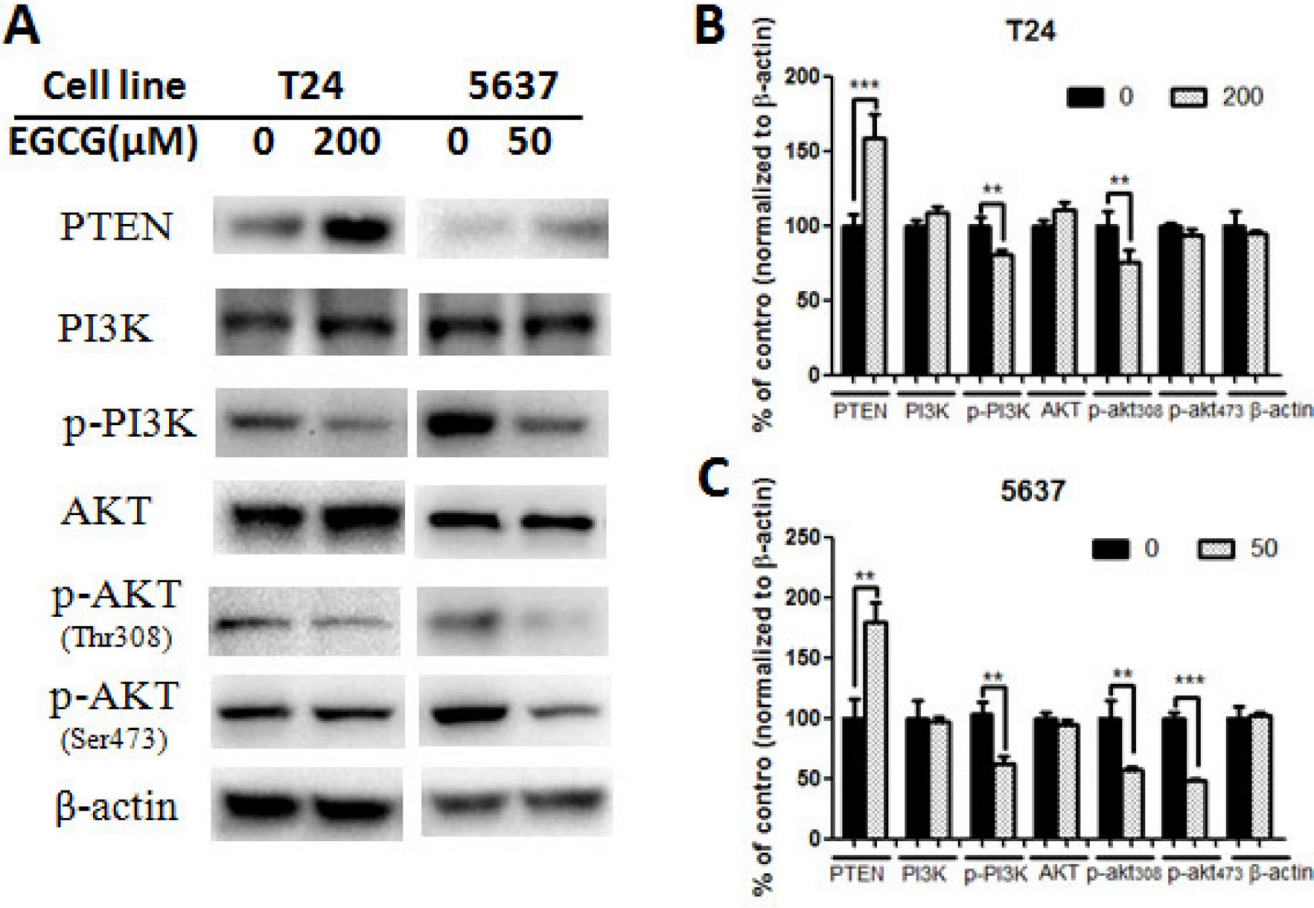
Effect of EGCG on protein expression of PI3K/AKT signaling pathway (**A**) Representative images of western blot of proteins after treated with EGCG in T24 and 5637 cells. (**B**–**C**) Statistical analysis of PTEN, PI3K, phosphorylated PI3K, AKT, phosphorylated AKT (Thr 308), phosphorylated AKT (Ser 473) and β-actin protein expressions in T24 (B) and 5637 (C) cells after EGCG treatment. Data were showed as mean + SD (*n* = 3). ^**^p < 0.01 and ^***^p < 0.001, as compared with untreated control.

Treatment with EGCG for 24 h resulted in the change of protein expression (Figures 4–5). EGCG-treated T24 and 5637 cells induced the cleavage of protein caspase-3 and PARP, and significant differences were shown between control and EGCG-treated group, indicating the apoptosis induction effects of EGCG in bladder cancer T24 and 5637 cells (Figure 4). No obvious difference was shown in NF-κB p65 and phosphorylated NF-κB p65 (p- NF-κB p65) in both T24 and 5637 cells. However, EGCG up-regulated the expression of PTEN. EGCG showed no obvious effect on PI3K and AKT expression, but decreased the phosphorylated PI3K and phosphorylated AKT (Thr308 and Ser473) expression in both T24 and 5637 cells (Figure 5), indicating EGCG inhibited T24 and 5637 cell proliferation and migration via modulation of PI3K/AKT pathway.

### EGCG decreased tumor burden in nude mice without obvious toxicity to the hosts

**Figure 6:**
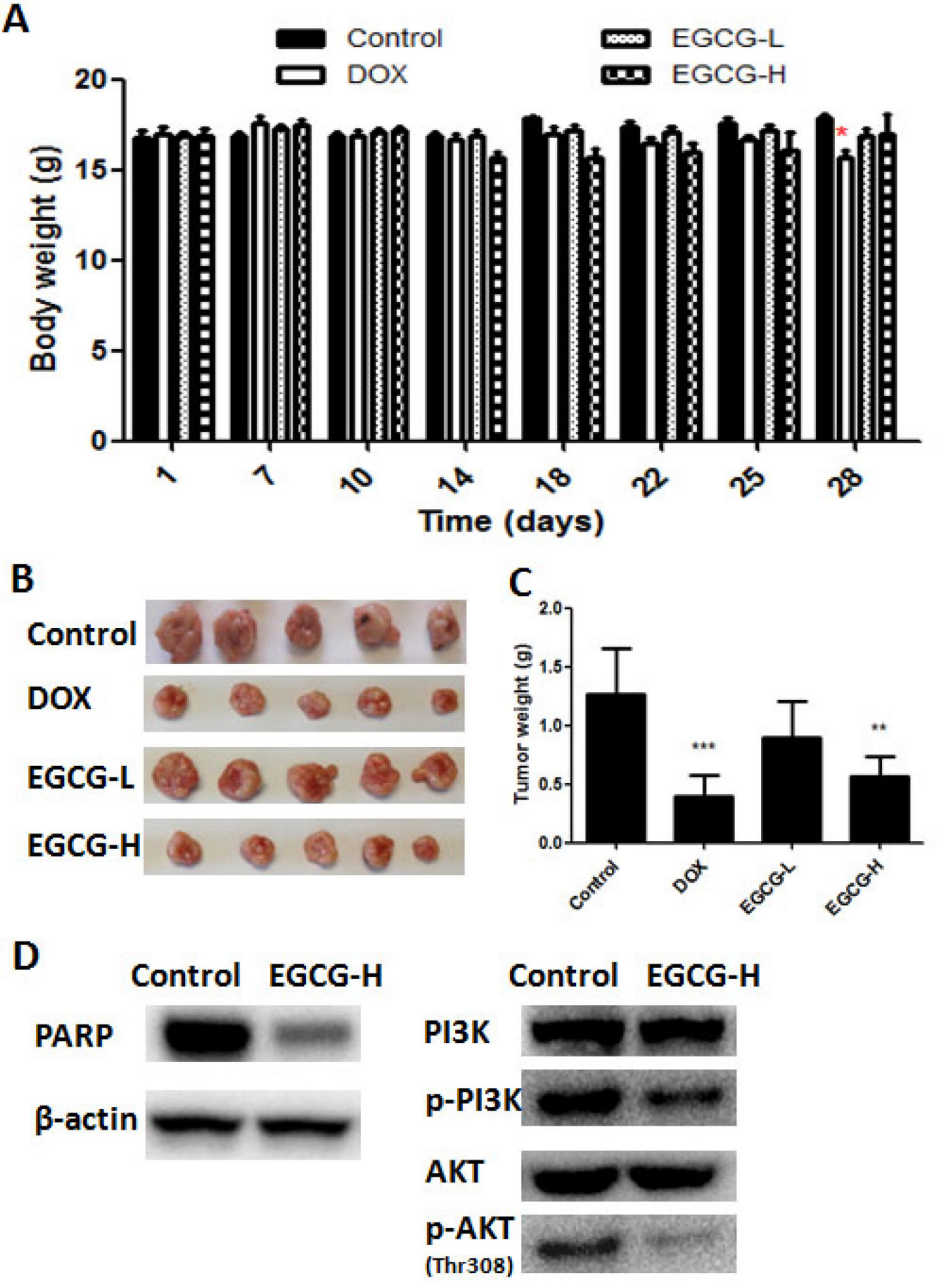
*In vivo* anti-tumor effect of EGCG in T24 nude mice xenograft tumor model (**A**) No significant body weight loss of mice was found in EGCG treatment groups. (**B**) Representative images of tumor from each group at the termination of the experiment. (**C**) Graph showed the tumor weight from different group. High dose EGCG treatment resulted in significant decrease in tumor weight. Data were expressed as mean +/ ± SEM, *n* = 7. ^*^p < 0.05 and ^**^p < 0.01, as compared with control. (**D**) Effect of EGCG on protein (PARP, PI3K, phosphorylated PI3K, AKT, phosphorylated AKT ) expression in tumor.

To investigate the activity of EGCG on tumor growth *in vivo*, a subcutaneous tumor model in nude mice was employed, in which cells were injected into the subcutis of BALB/c nude mice. It was observed that no significant body weight loss was found in EGCG-treated groups during the treatment, but significant body weight loss was found in positive control DOX group (Figure 6A). The tumor growth was inhibited in EGCG and DOX treatment groups, and EGCG-H and DOX decreased the tumor weight significantly by 51.2% and 63.8%, respectively (Figure 6B–6C). Besides, tumors were excised from nude mice to detect the protein expression. As shown in Figure 6D, treatment of EGCG (100 mg/kg) in nude mice resulted in obviously inhibition of phosphorylated PI3K and AKT, and activated PARP, this results were in line with the findings *in vitro.*

### EGCG showed no obvious effect on T24 and 5637 cell migration when AKT was inhibited

**Figure 7:**
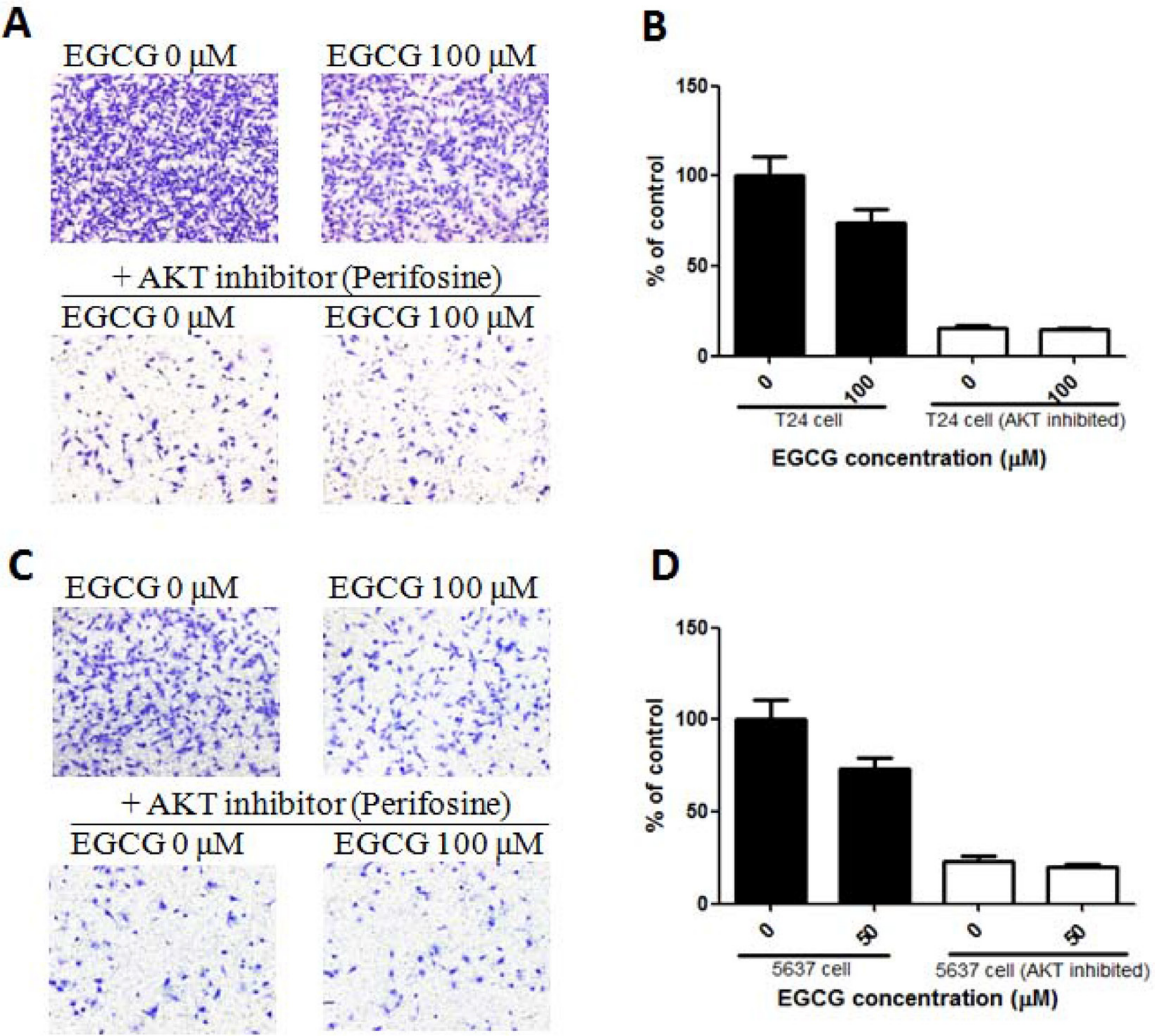
Effect of EGCG in normal and AKT inhibited bladder cancer cells (**A**) Representative images of the stained T24 and AKT inhibited T24 cells. (**B**) Anti-invasion effects of EGCG in normal and AKT inhibited T24 cells. (**C**) Representative images of the stained T24 and AKT inhibited 5637 cells. (**D**) Anti-invasion effects of EGCG in normal and AKT inhibited 5637 cells. Data were expressed as mean + SD, *n* = 3.

In order to confirm the important role of PI3K/AKT in EGCG induced proliferation and migration inhibition, AKT inhibitor 10 μM (Perifosine, Selleck) was added in T24 and 5637 cells, and then the cells were collected for transwell assay. As shown in Figure 7A, EGCG inhibited cell invasion in normal T24 cells, but no obvious effect was shown in AKT inhibited T24 cells. Similar result was also present in 5637 cells that EGCG induced significant inhibition of cell invision in 5637 cells, without obvious effect in AKT inhibited 5637 cells (Figure 7C–7D).

## DISCUSSION

Green tea is the popular and well-known beverage worldwide, especially in China, which is obtained from the dried leaves of the plant *Camellia sinensis*. Green tea is well documented to be effective in anti-obesity, anti-oxidation, cardiovascular protection and anti-cancer effects, and tea polyphenol EGCG is the most abundant and bioactive ingredient in green tea. Throughout the world, tea polyphenol especially EGCG has been widely served as health care products. Given the increasing popularity and commercial development of EGCG in cancer treatment, it is urgently needed to study the comprehensive protection of EGCG against bladder cancer. In this study, we aim to investigate the anti-proliferation and anti-migration effects of EGCG in bladder carcinoma T24 and 5637 cells both *in vitro* and *in vivo*.

In this study, we found that treatment of EGCG resulted in dose- and time-dependent inhibition of cell viability on both T24 and 5637 cells *in vitro*. The cytotoxicity of EGCG on normal human bladder epithelium SV-HUC-1 cells was also tested, and the IC_50_ in SV-HUC-1 cells was much higher than that in T24 and 5637 cells (Figure 1). Singh et al. elucidated that the tea polyphenol EGCG seemed to get quite specific chemopreventive effect, as EGCG has shown higher cytotoxicity in cancer cells than in their normal counterparts [20]. To determine whether the anti-proliferative effect of EGCG was associated with apoptosis induction, AV/PI double staining was employed. The results showed that EGCG induced apoptosis in T24 and 5637 cells in a dose-dependent manner (Figure 2). The findings were in line with the effect of EGCG on T24 and MBT-2 cells, which induced apoptosis in bladder cancer by activation of Bcl-2 family proteins [10–11]. Besides, *in vitro* studies elucidated that EGCG induced apoptosis in a variety of tumor cell lines, such as colon cancer, breast cancer, hepatoma and prostate cancer [20]. Apoptosis, a vital processes in normal cell turnover and chemical-induced cell death, is considered as protective mechanism against cancer development. EGCG was also demonstrated to be effective in inhibition of cell proliferation on T24, SW780 and TCCSUP cells [15, 21]. Apart from the anti-proliferation and apoptosis induction effects of EGCG, the non-cytotoxic dose of EGCG was found to be effective in inhibiting T24 and 5637 cell migration and invasion in a dose-dependent manner as assessed by wound healing and transwell migration assays (Figure 3).The results were comparable to previous reports that EGCG was effective in inhibition of proliferation and migration in bladder cancer SW780 cells, human breast cancer MDA-MB-231, MCF-7 cells and colon cancer SW620 cells [15, 22–24]. This non-cytotoxic does of EGCG showed significant anti-migration and anti-invasion effects to T24 and 5637 cells. That means EGCG could be added as a nutritional supplement for bladder cancer prevention and treatment.

To gain insight into the underlying mechanism of EGCG-induced apoptosis and migration inhibition, several proteins were tested, including NF-κB, caspases-3, PARP, PTEN, PI3K and AKT. EGCG was effective in activation of caspases-3 and PARP (Figure 4), indicating that EGCG induced apoptosis in both T24 and 5637 cells, which was in line with the findings of Annexin V/PI staining. The result was also in complete agreement with our previous finding that EGCG treatment resulted in down regulation of caspase-3 with a concomitant activation in PARP in SW780 cells [15]. The results suggested that EGCG-induced apoptosis played a vital role in the inhibition of T24 and 5637 cells, as apoptosis was considered as a protective mechanism against cancer development. Besides, EGCG showed no obvious effect in NF-κB p65 and phosphorylated NF-κB p65 in both T24 and 5637 cells. The finding was totally different from the results of EGCG in SW780. The possibly reason maybe that SW780 is in the early stage of bladder cancer, while T24 and 5637 are the advanced bladder cancer cells with high capacity of proliferation and metastasis. Then, we found that EGCG up-regulated the expression of PTEN, and significantly decreased the phosphorylated PI3K and phosphorylated AKT (Thr 308 and Ser473) in both T24 and 5637 cells (Figure 5). PTEN is a potent inhibitor of the PI3K/AKT pathway, and PTEN loss would be associated with aggressive tumor growth and metastases [25]. One study group actually reported that the PTEN expression was reduced in 94% patients with advanced stage of bladder cancer [17]. In addition, it was demonstrated that EGCG induced apoptosis in human pancreatic cancer cells via PTEN, and the nanoparticle form of EGCG up-regulation of PTEN in breast cancer MCF-7 cells [26–27]. Our results showed that EGCG was also effective in increasing the PTEN expression in bladder cancer T24 and 5637 cells. PI3K/AKT pathway plays an important role in cell metabolism, proliferation, apoptosis, and tumor development. Once AKT signaling was over activated, the cell proliferation, migration and angiogenesis would be promoted and also resulted in inhibition of apoptosis. It was demonstrated that AKT was activated in a wide variety of cancers including bladder cancer [28], and its activation results in enhanced resistance to apoptosis. We found that the phosphorylated PI3K and AKT were significantly down-regulated in both T24 and 5637 cells, and the apoptotic protein caspase-3 and PARP were significantly activated after EGCG treatment (Figure 5). The result was in line with Qin’s finding that EGCG inhibited PI3K/AKT activation, modulated Bcl-2 family proteins, and then resulted in enhanced apoptosis in T24 cells [11]. Similar findings were also shown in breast cancer, pancreatic cancer and hepatocellular carcinoma. Treatment of EGCG resulted in promoted apoptosis in human breast cancer T47D cells, pancreatic cancer PANC-1 cells and hepatocellular carcinoma SMMC7721 cells through modulation of PI3K/AKT signaling [29–31].

Apart from the *in vitro* studies, the anti-tumor effect of EGCG was investigated in a subcutaneous T24 tumor model. After treatment, no significant difference was shown on body weight in EGCG-treated groups, while the positive control (DOX) group induced significant body weight loss in mice at day 28. The result indicated that the treatment dose of EGCG was safe and showed no obvious toxicity to the hosts (Figure 6A). The administration of EGCG in high dose (EGCG-H) was able to decrease tumor weight significantly in mice bearing T24 tumors (Figure 6B–6C), which was in line with Hsieh’s finding that nanoparticle form of EGCG resulted in significant reduction of tumor weight in C3H/He nice with MBT-2 bladder tumor no matter in oral, intraperitoneal or intratumor mode of administration [12]. Kemberling et al. elucidated that instillation of EGCG resulted in significantly inhibition of AY-27 bladder tumor growth in Fisher rats [13]. Our previous study also demonstrated that EGCG decreased the SW780 tumor growth significantly [15]. In addition, a recent preclinical study revealed that EGCG prevented intravesical tumor growth in female Fischer rats [32]. A clinical investigation from Liu et al. demonstrated that intravesical irrigation of EGCG in patients with interstitial cystitis resulted in remission of symptoms, such as attenuated the expression of purinergic receptors and up-regulation of iNOS [33]. Clinical studies from breast cancer also demonstrated that treatment with EGCG or tea polyphenol catechin resulted in significant inhibition of tumor growth in patients [34]. Besides, treatment with EGCG high dose group (EGCG-H) in nude mice resulted in notable activation of PARP, and significant inhibition of phosphorylated PI3K and AKT in tumor (Figure 6D). The *in vivo* findings in tumor were completely in line with the *in vitro* result that EGCG significantly suppressed phosphorylated PI3K and AKT expression. Furthermore, when AKT was inhibited, EGCG showed no obvious effect in T24 and 5637 cell migration (Figure 7), indicating the important role of AKT signaling played in EGCG induced effect. PI3K/AKT pathway plays a crucial role in cell metabolism, apoptosis and tumor development. PTEN loss or AKT over activated could resulted in apoptosis inhibition and promoted cell proliferation and migration and then regulates the expression of a large number of proteins, including BAD, caspase-3 and tumor suppressor p53 [35]. Our results demonstrated that EGCG was effective in decreasing the expression of phosphorylated PI3K and AKT both *in vitro* and *in vivo*, and showed significant anti-proliferation and anti-migration effects in T24 and 5637 cells. This revelation sheds light on the underlying mechanisms of EGCG on tumor inhibition in bladder cancer T24 and 5637 cells, and provides clear directions for cancer treatment and drug combination.

Our study demonstrated that the dosage of EGCG was effective in anti-tumor in mice in mice bearing bladder tumor, without obvious toxicity to the hosts as assessed by body weight, which highlighted EGCG as a natural potential drug in prevention and treatment of cancer. The effective dose of EGCG (100 mg/kg) was equivalent to a single dose of 487 mg EGCG powder for a 60 kg adult. This high concentration of EGCG was unlikely to be consumed from tea beverage or food intake, but it would be achievable for human beings to consume the effective dose of EGCG as a powder capsule in concentrated form. In this regards, similar dose of EGCG was demonstrated by Henning et al. that the single dose of purified EGCG (518 mg) was healthy for individuals in anti-oxidant [8]. Besides, Zhang et al. elucidated that EGCG in 400 mg capsules orally administered three times per day to breast cancer patients undergoing radiotherapy resulted in significantly lower serum levels of VEGF, HGF, MMP9 and MMP2, when compare to patients who received radiotherapy alone [36].

In conclusion, our results present evidence on the anti-tumor and anti-proliferation effects of EGCG against T24 and 5637 via modulation of PI3K/AKT pathway. More detailed molecular mechanisms, for instance, genomic and proteomic responses underlying the EGCG-induced bladder cancer cell apoptosis and anti-metastasis remain to be elucidated. The pharmacokinetics of EGCG in human beings also needs investigation in future. Besides, further investigation is needed to determine the clinical efficacy and safety of EGCG in human subjects with bladder cancer. Our observation holds promise for further studies to examine the efficacy of EGCG and develop EGCG as a potential anti-cancer supplement against bladder cancer.

## MATERIALS AND METHODS

### Cells and reagents

The T24 and 5637 human bladder cancer cells were cultured in DMEM medium and RPMI-1640 medium respectively, containing 10% (v/v) fetal bovine serum and 1% penicillin-streptomycin (Life technology, USA) at 37°C in 5% CO_2_ humidified incubator. Cell Counting Kit-8 (CCK8) was obtained from Trans Gen Biotech, China. Transwell plates were from Corning Incorporated, USA. Caspase-3, PARP, NF-κB p65, p-NF-κB p65, PI3K, p-PI3K, AKT, p-AKT (Thr 308, Ser473) were purchased from Cell signaling technology, USA. Perifosine, AKT signaling pathway inhibitor, was provided by Selleck, USA.

### Cell viability assay

T24 or 5637 cells were placed in 96-well culture plates at 1 × 10^5^ cells/ml with medium containing 10% FBS. After 24 h incubation, cells were treated with different concentrations of EGCG for 24 h and 48 h. Following incubation, 10 µl of CCK8 solution was added to each well and the plate was incubated at 37°C for another 2 h in dark. The Absorbance was analysed using aenzyme-labeled instrument in 450 nm (ThermoMultiskan GO, USA).

### Annexin V-FITC/PI double staining

After treatmented with EGCG, T24 or 5637 cells were collected and washed twice with ice-cold PBS. Then, Annexin V-FITC and PI were added to each sample and incubated in dark for 15 min at room temperature. The fluorescent signal in cells was analyzed by flow cytometry (FACSARIA II, Becton Dickinson) within 1 h. Positioning of quadrants on annexin-V/PI plots was performed to distinguish living cells (FITC-/PI-), early apoptotic cells (FITC+/PI-) and late apoptotic or necrotic cells (FITC+/PI+).

### Scratch wound healing assay

The efficacy of EGCG on cell migration was assessed using scratch wound healing assay. Firstly, T24 or 5637 cells were plated at 1 × 10^5^/well cells in24-well plates. After starved in medium without fetal bovine serum for 24 h, cells were scraped with crosses using a sterile plastic tip, and then the original medium was replaced the fresh medium with preselected concentration of EGCG. Then, the plate was incubated for 24 h, wound healing was observed and photographed under a microscope (Olympus IX73). The percentages of open wound area were measured and calculated using the TScratch software.

### Transwell migration assay

During the transwell migration assay, cells at 2 × 10^5^/ml were added in the upper transwell migration chamber and cultured in 200 µl 1% v/v FBS medium containing various concentrations of EGCG. Then 500 µl complete medium (with 10% v/v FBS) was added in the lower well of 24-well plates as chemoattractant media. After incubated for 24 h at 37°C, cells were fixed with methanol and stained with 0.1% crystal violet. Stained filters were photographed under microscope (Olympus IX73). The migrated cells were quantified by manual counting and represented as a percentage of control values.

### Western blot analysis

T24 or 5637 cells treated with EGCG for 24h were collected and lysed in lysis buffer (50 mM Tris-HCL, 0.5mM EDTA, 1% SDS, 1mM DTT) on ice. After the lysate boiled, protein samples (20 µg) were fractionated in 10% SDS-polyacrylamide gel and then transferred to PVDF membrane (Millipore, USA). Membranes were blocked with 10% non-fat milk and washed with PBS-T, and then incubated with primary antibody (dilution at 1:1000) at 4°C for 2 h. After washed with PBS-T, membranes were incubated with secondary antibodies conjugated with horseradish peroxidase for 1 h. Finally, visualization of protein bands was performed using the ECL substrate reagent kit (GE Healthcare) on a Gel Doc XR imaging system (Bio-RAD, USA).

### Animal study

Female BALB/c mice (6–8 weeks of age) were provided by Vital River Laboratory Animal Technology Co. Ltd, Beijing, and were housed under pathogen-free conditions in Shenzhen Institutes of Advanced Technology (Licence: SYXK 2012–0119), Chinese Academy of Sciences. T24 cells (2 × 10^6^) resuspended in 0.2 ml PBS, were subcutaneously (s.c.) inoculated at the back of each mouse. After T24 cell implantation, the tumor-bearing mice were randomly assigned into four groups (*n* = 6): Control group (saline, i.p. injected everyday), Doxorubicin (DOX) group (2 mg/kg DOX, i.p. injected once, served as positive control), EGCG low (EGCG-L) dose group (50 mg/kg EGCG, i.p. injected everyday) and EGCG high (EGCG-H) dose group (100 mg/kg EGCG, i.p. injected every day). Treatments were initiated one week after cancer cell implantation and lasted for four weeks. During treatment, the body weight of each mouse was measured twice a week. At day 28, mice were sacrificed, and the tumors were removed for quantification of tumor burden. The tumors in EGCG high dose group were lysed for analysis of protein expression.

### Statistical analysis

Each experiment was performed in three times and all data in graph were expressed as mean ± SD. Statistical differences were calculated by the one-way analysis of variance (ANOVA), with *p* < 0.05 was considered as statistically significant.
